# Systolic blood pressure increase in chronic heart failure associates with survival advantage

**DOI:** 10.1097/j.pbj.0000000000000284

**Published:** 2025-03-18

**Authors:** Helena Rocha, Rita Gouveia, Catarina Elias, Catarina Reis, Ana Margarida Fonseca, Adriana Costa, Carolina Guimarães, Rui Ribeiro, Ana Toste, Carlos Grijó, Helena Reis, Ana Neves, Jorge Almeida, Patrícia Lourenço

**Affiliations:** a Internal Medicine Department, Centro Hospitalar de São João, Porto, Portugal; b Department of Medicine, Faculty of Medicine, Porto University, Porto, Portugal

**Keywords:** systolic blood pressure variation, chronic heart failure, paradox, mortality

## Abstract

**Background:**

The impact of systolic blood pressure (SBP) variation on chronic heart failure (HF) is largely unknown. We assessed the impact of SBP variation in patients with chronic HF.

**Methods:**

This is a retrospective analysis of adult ambulatory patients with HF with left ventricular systolic dysfunction (LVSD). SBP variation = SBP at the index visit – SBP at the 1-year visit. Patients dying in the first year or with missing data concerning SBP were excluded. Patients with SBP increase ≥10 mmHg during the first year were compared with the remaining. Determinants of SBP increase were assessed by binary logistic regression analysis. The patients were followed up from the 1-year visit up to 5 years. The primary end point was all-cause mortality. A Cox regression analysis was used to determine the association of SBP variation with mortality.

**Results:**

We studied 787 patients (68% male), with a mean age of 70 years. SBP increased by ≥10 mmHg in 277 patients (35.2%) and remained stable or decreased in 510. Patients in whom SBP increased more often presented severe LVSD and nonischemic HF; they had lower baseline SBP and were more medicated with loop diuretics. Independent predictors of SBP increase were lower basal SBP and loop diuretic use. Patients with a SBP increase ≥10 mmHg had a crude hazard ratio (HR) of all-cause mortality of 0.74 (0.59–0.94), and the multivariate-adjusted HR was 0.61 (0.46–0.79).

**Conclusions:**

Patients with chronic HF with SBP increase ≥10 mmHg over the first year have a 39% reduction in the all-cause mortality risk irrespective of basal SBP, severity of ventricular dysfunction, and evidence-based drug use. Patients with SBP stability or decrease have a similarly poor prognosis.

## Introduction

Blood pressure targets have been difficult to define and are still evolving in the general and hypertensive populations.^1-4^ If this is true for the general and hypertensive patients, it is even more true for patients with heart failure (HF) whether they have hypertension history, or not. Particularly in patients with HF with reduced ejection fraction, hypertension may no longer be a problem and hypotension may surge as an expected side effect of evidence-based drugs used to modify HF prognosis.^5^

A J curve association of blood pressure with poor outcome and mortality in hypertensive patients alerts us not to lower blood pressure below values that increase the risk of myocardial infarction and death^6-8^; however, in general, higher systolic blood pressure (SBP) associates with poorer outcome.^9^ On the contrary, in patients with HF, whether acute or chronic, low blood pressure predicts more ominous outcome^10,11^ and higher blood pressure associated with better outcomes.^10,12^ This finding is called blood pressure paradox and renders HF therapy titration a challenge. In 2009, in a meta-analysis of 10 different studies reporting the association of blood pressure with mortality in patients with chronic stable HF, Raphael et al^13^ demonstrated 13.0% lower mortality with a 10-mmHg higher SBP.

In acute HF, an early blood pressure drop has been reported to correlate with higher mortality.^14,15^ Cotter et al^14^ alerted for the cautious administration of vasodilating agents to patients with acute HF, especially when significant decrease in systolic blood pressure is observed. The prognostic value of changes in blood pressure has been addressed in chronic HF, and patients with more stable blood pressure values appear to have survival advantage.^16-19^ However, the impact of a decrease or rise in systolic blood pressure on chronic HF over a determined time interval, more than its visit-to-visit variation, is largely unknown.

We aimed to study the prognostic implication of systolic blood pressure variation during a 12-month period.

## Methods

We retrospectively analyzed adult outpatients with chronic HF with left ventricular systolic dysfunction (LVSD) followed in a HF clinic from January 2012 to December 2020. The index visit was considered the first patient evaluation since January 2012. The diagnosis of HF was based on updated guidelines of the European Society of Cardiology by the time the patients were evaluated.^20,21^ The HF phenotype was based on the most recent guidelines^5^: patients with ejection fraction between 41% and 49% were classified as having HF with mildly reduced ejection fraction (HFmrEF) and those with ejection fraction ≤40% as having HF with reduced ejection fraction (HFrEF); within patients with HFrEF, those with ejection fraction ≤30% were considered as having severe systolic dysfunction. The diagnostic echocardiogram was performed peri index visit: upon referral for patients referred from the hospital; and for patients referred from the primary care, an echocardiogram was performed in the index visit or within approximately 3 months. Demographic data and data concerning comorbidities, physical examination, laboratory parameters, and medication at the end of the index visit were abstracted from the patients' electronic files.

Patients with blood pressure measurement both in the index visit (first visit) and in the 1-year visit were included in the study. Patients dying in the first year of the HF clinic attendance or with missing data concerning SBP were excluded from the analysis. A standard sphygmomanometer or, more frequently, a validated blood pressure–measuring device, was used to measure blood pressure. Measurements were made in the sitting position. When using a sphygmomanometer, measures were read to the nearest 2 mmHg and the Korotkoff phase V sounds were used as the criterion for diastolic blood pressure. SBP variation was calculated as SBP at the index visit − SBP at the 1-year visit.

The end point under analysis was all-cause mortality, and vital status was assessed by consulting hospital registries and by telephone contact with the patients or their relatives. When no information was obtained, we consulted the *Registo Nacional de Utentes* platform. Patients were followed up to 5 years until January 2023. Follow-up was set since the 1-year follow-up visit onward. No patient was lost to follow-up.

The study protocol conforms to the ethical guidelines of the Declaration of Helsinki, and it was approved by the CHUSJ ethics committee. Owing to the retrospective nature of the design, informed consent was not signed.

### Statistical analysis

Categorical variables are presented as counts and proportions. Continuous variables are presented as mean ± standard deviation when normally distributed and median (interquartile range) when non-normally distributed. The comparison between basal and 1-year SBP was made using a Wilcoxon rank-sum test.

Patients were classified into 3 groups: those with ≥10 mmHg SBP decrease, patients with SBP within ±10 mmHg variation, and those with ≥10 mmHg increase during the first year. Survival curves were determined by the Kaplan–Meier method. Since patients with stable SBP or decrease ≤10 mmHg were similar concerning prognosis, they were gathered in a single group. Patients with SBP increase of 10 mmHg and above were compared with the remaining using the chi-square test for categorical variables, the Student *t* test for normally distributed continuous variables, and the Mann–Whitney U test for continuous variables with non-normal distribution.

A binary logistic regression analysis was used to assess independent determinants of SBP increase. A multivariate model was built based on variables associated with SBP increase in a univariate approach. Evidence-based drugs, with known hypotensive effect, were also considered in the model.

Patients were followed from the 1st year visit until January 2023, and the primary end point under analysis was all-cause mortality. A Cox regression analysis was used to determine the association of SBP variation with all causes of mortality. A multivariate analysis was conducted adjusting for potential confounders. Models of increasing complexity were built: model 1 accounted for age, sex, major comorbidities, severity of LVSD, basal systolic and diastolic blood pressure, and New York Heart Association (NYHA) class and model 2 included variables in model 1 and basal hemoglobin, B-type natriuretic peptide (BNP) and estimated glomerular filtration rate, loop diuretic use, and evidence-based drug use.

The *P* value considered for statistical significance was 0.05. Data were stored and analyzed using SPSS software (IBM corp, Armonk, NY, version 29.0).

## Results

**Figure 1.**
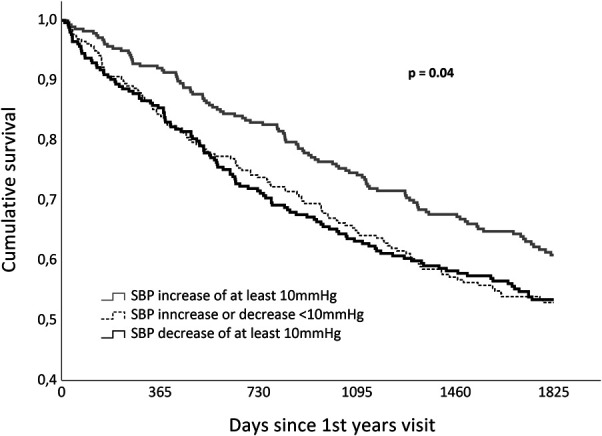
Kaplan–Meier survival curves according to systolic blood pressure variation over the first year. Patients with systolic blood pressure increase present survival advantage over the remaining groups. Patients with systolic blood pressure decrease have similar prognosis than those with blood pressure stability.

We included 787 patients (68% male), with a mean age of 70 years. Patient characteristics are given in Table 1. The mean systolic blood pressure at the index visit was 121 (±21) mmHg, and at 1-year visit, it was 121 (±21) mmHg. There was no significant difference between basal and 1-year SBP (*P* = .93), and the median (interquartile range) SBP variation was 0 (−14 to +14) mmHg (percentile 33.3 = −10 mmHg and percentile 66.7 = 8 mmHg increase). The SBP increased by ≥10 mmHg in 277 patients (35.2%); it decreased by ≥10 mmHg in 254 (32.3) and remained stable in 256 (32.5%). Figure 1 shows the Kaplan–Meier curves in these 3 groups of patients. Patients with SBP increase of at least 10 mmHg had a clear survival advantage. The groups with stable and decreasing SBP had a similarly ominous outcome.

**Table 1:** Patients' characteristics and comparison between groups of patients with SBP increase ≥10 mmHg over the first year and the remaining

Characteristics	All (n = 787)	SBP increase ≥10 mmHg (n = 277)	SBP stability or decrease ≥10 mmHg (n = 510)	*P*
Male sex, n (%)	535 (68)	179 (64.6)	356 (69.8)	.14
Age, mean (SD)	70 (13)	68 (14)	70 (12)	.08
SBP at baseline, mean (SD)	121 (21)	112 (17)	126 (21)	<.001
DBP at baseline, mean (SD)	67 (12)	63 (10)	68 (12)	<.001
Heart rate, mean (SD)	74 (14)	74 (13)	75 (14)	.90
Diabetes *mellitus*, n (%)	295 (37.5)	108 (39.0)	187 (36.7)	.52
Hypertension, n (%)	453 (57.6)	158 (57.0)	295 (57.8)	.83
Atrial fibrillation, n (%)	267 (34.0)	93 (33.6)	174 (34.1)	.88
Ischemic etiology, n (%)	345 (43.8)	110 (39.7)	235 (46.1)	.09
NYHA class ≥ II, n (%)	497 (63.2)	171 (61.7)	326 (63.9)	.54
Severe systolic dysfunction, n (%)	373 (47.4)	145 (52.3)	228 (44.7)	.04
Hemoglobin (g/dL), mean (SD)	13.2 (1.8)	13.0 (1.8)	13.4 (1.8)	.03
eGFR (mL/min/1.73 m^2^), mean (SD)	73 (30)	73 (30)	73 (30)	.93
BNP (pg/mL), median (IQR)	249.4 (105.7–484.8)	246.3 (102.2–458.9)	250.1 (107.3–5111.5)	.82
Beta-blockers, n (%)	731 (92.9)	257 (92.8)	454 (92.9)	.93
ACEi/ARB/ARNi, n (%)	684 (86.9)	234 (84.5)	450 (88.2)	.14
MRA, n (%)	229 (29.1)	88 (31.8)	141 (27.6)	.22
Loop diuretics, n (%)	627 (79.7)	237 (85.6)	390 (76.5)	.002
All-cause mortality, n (%)	336 (42.7)	104 (37.5)	232 (45.5)	.03
Follow-up (days), median (IQR)	1671 (778–1825)	1761 (979–1825)	1576 (643–1825)	.01

ACEi, angiotensin receptor inhibitor; ARB, angiotensin receptor blocker; ARNi, angiotensin receptor/neprilysin inhibitor; BNP, B-type natriuretic peptide; eGFR, estimated glomerular filtration rate; IQR, interquartile range; NYHA, New York Heart Association; SBP, systolic blood pressure.

Patients with SBP increase in the 1st year had significantly lower systolic and diastolic blood pressure and higher prevalence of severe LVSD and lower basal hemoglobin and were more often medicated with loop diuretics. They were, however, similar concerning comorbidity burden, basal NYHA class, evidence-based drug use, and basal BNP and renal function. Despite this similarity, apart from lower basal blood pressure and poorer left ventricular function, they had significantly lower mortality (37.5 vs 45.5%, *P* = .03) in a longer follow-up period (Table 1).

During follow-up, 336 patients (42.7%) died. The independent prognostic impact of SBP increase of at least 10 mmHg was assessed by multivariate Cox regression analysis, as given in Table 2. Patients with at least 10-mmHg increase in SBP during the first year had a hazard ratio (HR) of all-cause mortality up to 5 years of 0.67 (0.53–0.86, *P* = .002) in model 1 and of 0.61 (0.46–0.79, *P* < .001) in model 2 (Table 2). It is important to note that in the multivariate analysis, lower basal SBP also portended worse prognosis [HR = 1.11 (95% CI: 1.03–1.20) per 10 mmHg decrease in SBP at the index visit], as well as older age, diabetes, atrial fibrillation, lower hemoglobin, and higher BNP.

**Table 2:** Association of systolic blood pressure increase ≥10 mmHg with all-cause mortality: crude and multivariate analyses

SBP increase ≥10 mmHg	HR (95% CI)	*P*
Crude	0.74 (0.59–0.94)	.01
Multivariate-adjusted model 1*	0.67 (0.53–0.86)	.002
Multivariate-adjusted model 2†	0.61 (0.46–0.79)	<.001

* Adjustments for age, sex, arterial hypertension history, diabetes *mellitus,* atrial fibrillation, ischemic heart failure, severe left ventricular systolic dysfunction, basal systolic and diastolic blood pressure, and NYHA class ≥II.

† Adjustments for variables considered in model 1 + basal hemoglobin; estimated glomerular filtration rate and B-type natriuretic peptide; loop diuretic use; and treatment with renin–angiotensin–aldosterone inhibitors, mineralocorticoid receptor antagonists, and beta-blockers.

The only independent predictors of SBP increase ≥10 mmHg were lower SBP at the index visit with an OR of 0.69 (95% CI: 0.61–0.77) per each 10 mmHg increase in basal systolic blood pressure (*P* < .001) and loop diuretic use with an OR of 1.88 (95% CI: 1.19–2.96, *P* = .007). The predictors of SBP increase in both univariate and multivariate approaches are given in Table 3.

**Table 3:** Predictors of SBP increase of at least 10 mmHg during the first year in patients with HF: binary logistic regression analysis

	Univariate analyses		Multivariate analyses	
	OR (95% CI)	*P*	OR (95% CI)	*P*
Male sex	0.79 (0.58-1.08)	.14		
Age, per 10-year increase	0.91 (0.81-1.02)	.09	0.89 (0.77–1.02)	.10
Basal SBP, per 10-mmHg increase	0.67 (0.61-0.73)	<.001	0.69 (0.61–0.77)	<.001
Basal DBP, per 10-mmHg increase	0.69 (0.60-0.79)	<.001	0.94 (0.78–1.14)	.53
Basal heart rate, per 10-bpm increase	1.02 (0.97-1.08)	.34		
Diabetes *mellitus*	1.10 (0.82-1.49)	.52		
Arterial hypertension	0.97 (0.72-1.30)	.88		
Atrial fibrillation	0.98 (0.72-1.33)	.88		
Ischemic HF etiology	0.77 (0.57-1.04)	.09	0.88 (0.63–1.24)	.48
NYHA class ≥II	0.91 (0.67-1.23)	.54		
Severe LVSD	1.36 (1.01-1.82)	.04	0.98 (0.70–1.37)	.91
Basal hemoglobin, per each 1 g/dL increase	0.91 (0.84-0.99)	.03	0.96 (0.88–1.06)	.43
Basal eGFR, per each 10 mL/min/1.73 m^2^	1.00 (0.95-1.05)	.94		
Basal BNP, per 100-pg/mL increase	1.01 (0.98-1.03)	.60		
Loop diuretics at the end of the 1st visit	1.82 (1.23-2.79)	.003	1.88 (1.19–2.96)	.007
RASi at the end of the 1st visit	0.73 (0.48-1.11)	.14	0.87 (0.54–1.40)	.56
MRA at the end of the 1st visit	1.22 (0.89-1.68)	.22	0.90 (0.63–1.30)	.58
Beta-blocker at the end of the 1st visit	0.98 (0.79-1.21)	.93	1.07 (0.57–1.99)	.84

BNP, B-type natriuretic peptide; bpm, beats per minute; CI, confidence interval; DBP, diastolic blood pressure; eGFR, estimated glomerular filtration rate; HF, heart failure; LVSD, left ventricular systolic dysfunction; MRA, mineralocorticoid receptor antagonist; NYHA, New York Heart Association; OR, odds ratio; RASi, renin–angiotensin–aldosterone system inhibitor; SBP, systolic blood pressure.

## Discussion

In our cohort of 787 ambulatory patients with chronic HF with systolic dysfunction, we reproduce the blood pressure paradox as, per 10 mmHg increase in basal SBP, there is a 10% (95% CI: 3–17%) reduction in the risk of all-cause mortality up to 5 years and we further reinforce it by showing that patients with an increase of at least 10 mmHg over a 1-year period have a multivariate-adjusted mortality reduction of 39% (95% CI: 21%–54%) in the upcoming 5 years. The only independent predictors of SBP increase ≥10 mmHg over 1 year were a lower basal systolic blood pressure and the need of loop diuretic dose to manage congestion. Neither the severity of LVSD nor the use of evidence-based drugs were independent predictors of SBP increase. Importantly, the association of SBP increase of at least 10 mmHg over a 1-year period with survival benefit was independent of basal systolic blood pressure and of symptomatic and prognostic modifying drugs.

In the acute HF setting, an early (24–48 hours) drop in SBP was reported to be associated with poor prognosis,^14,15,22,23^ and this has raised concerns regarding the aggressive or eventually untimely use of drugs with potential hypotensive action. While the SBP drop has been suggested as potentially harmful in the acute setting, no such analysis has ever been performed in the chronic setting. Evidence-based drugs are to be titrated to target doses; however, hypotension is frequently the limiting factor in such strategy. How should we interpret the SBP trajectory in ambulatory patients with stable HF? Changes in blood pressure over time in chronic HF have been mainly addressed by analyzing SBP variability. Multiple blood pressure measurements over time have been used to calculate the visit-to-visit blood pressure variability: standard deviation of the mean of the patients' blood pressure measurements; the coefficient of variation calculated as the standard deviation divided by the mean blood pressure of all measurements; or the average absolute difference of several sequential measurements along follow-up visits. A higher SBP variability, over −10 or +10 mmHg/year based on multiple SBP measurements over time, was reported to independently predict mortality in patients with chronic HF, the patients with the most stable blood pressure measurements being the group with best survival. This correlation of higher SBP variability with worse outcome was reported for patients with HF with reduced^17,18^ and preserved ejection fraction.^16,19,24^ This association of higher BP variability with mortality was also found in general population^25^ and in hypertensive patients.^26^ In chronic HF, a decreased short-term BP variability is viewed as an index of sympathetic vascular modulation and is associated with increased mortality.^27,28^ The association of high SBP variability over long-term periods with adverse effects in patients with diabetes or hypertension has been interpreted as a result of increased shear stress, endothelial damage, microvascular lesions, coronary atheroma progression, and major bleeding, all of which can increase the risk of all-cause death,^29,30^ and these factors have been suggested to participate in the higher all-cause mortality in patients with HFpEF.^19^

The determination of blood pressure variability in clinical practice implies the availability of several measurements over time and a somehow complex mathematical calculus, making it more demanding for physicians. On the contrary, the measurement of variation of blood pressure between 2 definite points in time is a more amenable method. With such an approach, we found that patients with SBP increase of at least 10 mmHg in the first year of follow-up have a 39% reduction in all-cause mortality, irrespective of admission blood pressure, severity of LVSD, and evidence-based drug use. In the past 3 decades, chronic HF management focused on titrating, to maximum tolerated doses, the different types of drugs that have demonstrated to improve mortality, while some of them may have hypotensive effect.^5^ Importantly, although we reinforce the HF paradox in that, per 10 mmHg increase in basal SBP, there is a 10% reduction in all-cause mortality, the group of patients with SBP decrease of at least 10 mmHg over 1-year had similar prognosis when compared with those with SBP stability, suggesting that the occurrence of SBP decrease should not worry clinicians or deter them from titrating HF medication if the patients are asymptomatic. In the acute context, an early drop should be viewed as worrisome, but, apparently, not in the chronic context. In chronic HF, the blood pressure paradox is reinforced by the fact that an increase in SBP associates with better survival. Since the SBP in patients with HF, unlike in the general population, is more closely governed by the cardiac output than peripheral resistance,^13^ it is possible that this SBP increase is a result of systolic function recovery and improved cardiac output and ejection fraction. However, data on systolic function at the end of the first year were not widely available and not considered in the analysis.

It is essential to note that concerning independent determinants of SBP increase, patients with lower basal SBP showed higher probability of recovery, as well as those needing loop diuretic therapy. This favors the hypothesis that the existence of congestion amenable to address at presentation may be determinant for unloading the heart and increasing its efficiency. The basal heart rate was similar in patients irrespective of their SBP trajectory, and therefore, we can assume that SBP increase improved the heart's efficiency in increasing cardiac output. The independent association of lower SBP with SBP recovery may be purely mathematical; if patients have lower basal SBP, they have wider margin for recovery. It should be stressed that in the multivariate analysis, both lower SBP and absence of SBP increase were predictors of poor outcome notwithstanding lower SBP being a predictor of SBP increase.

Our study has obvious and major limitations. The retrospective nature and single-center small sample size were the most remarkable. Another important setback is the fact that no registry exists concerning the blood pressure measurement method nor the arm used for the measurements. This implies that different methods could have been used in the 2 time points, and that the blood pressure may have been measured in a different arm. The results would be easier to interpret if the same method and the same arm were used, and we cannot adjust for such a confounder. However, this is, in fact, what happens most of the time in clinical practice and, therefore, it reflects real-world treatment of patients with HF. Since the study concerns patients assessed between 2012 and 2020, only a small percentage of the patients taking renin–angiotensin system inhibitors were under angiotensin receptor neprilysin inhibitors; in addition, no patients were using sodium glucose transporter inhibitors for HF treatment. Furthermore, the fact that only basal and first-year SBP measurements were considered implies that the most severe patients, dying in the first year, were not considered in the analysis. It would be important to reproduce our results in a prospective cohort, with blood pressure measured using the same method, in the same arm, and probably in a shorter period of 3 or 6 months. As previously stated, it would have been important to have data on systolic function in the 1-year follow-up visit to better understand the association of SBP increase with mortality.

Despite the limitations mentioned, we believe that this is the first study addressing SBP trajectory in chronic ambulatory HF based on 2 measurements in the long term. Blood pressure is easy to measure and available everywhere and is of paramount clinical importance in HF management. Our results suggest that with only 2 SBP measurements in 2 different time points, we can stratify ambulatory patients with HF in risk categories.

## Conclusions

Patients with chronic HF with SBP increase of at least 10 mmHg over the first year of treatment have a 39% reduction in the all-cause mortality risk irrespective of basal SBP, severity of left ventricular dysfunction, and evidence-based drug use. Patients with SBP stability or decrease have a similarly poor prognosis.
